# Management of Advanced Adult Soft Tissue Sarcoma

**DOI:** 10.1080/13577140310001607266

**Published:** 2003-06

**Authors:** Vivien H. C. Bramwell

**Affiliations:** Department of Medicine Tom Baker Cancer Centre 1331 – 29th Street N.W. Alberta Calgary T2N 4N2 Canada


*Sarcoma*, June 2003, *VOL. 7, NO. 2, 43–55*

Taylor & Francis healthsciences

**REVIEW**

### Management of advanced adult soft tissue sarcoma

VIVIEN H.C. BRAMWELL

*Department of Medicine, Tom Baker Cancer Centre, 1331 – 29th Street N.W., Calgary, Alberta, T2N 4N2 Canada*

### Introduction

The rare incidence and heterogeneous nature of soft tissue sarcomas (STS) are formidable barriers to the conduct of large randomised controlled trials (RCTs). A search of computer databases, using a Cochrane optimal search strategy 1966–1996, yielded less than 100 RCTs investigating the management of bone and soft tissue sarcomas (Bramwell 1997, Proceedings 5th Annual Cochrane Colloquium – unpublished data). Of these, approximately 40 are relevant to the management of advanced/metastatic STS, but some report on treatments that are no longer used. Thus, recommendations for management of advanced STS, in a substantial proportion of situations, have to be based on findings from observational/phase II studies and/or clinical consensus, as better evidence does not exist.

### Definition of advanced sarcoma

STS arise from mesenchymal tissue which is ubiquitous in the body. In contrast with many cancers that relate to a particular site (e.g. breast) and that display a limited number of characteristic histologies (e.g. adenocarcinoma), STS are markedly heterogeneous in location and histology, and thus in behaviour. Advanced STS may be defined under two headings: (1) locoregional disease; (2) distant metastases.

### *(1) Locoregional disease*

There are three main situations in which locoregional disease becomes difficult to control, and may be life-threatening in the absence of distant metastases.

*Curative local treatment is impossible because of location*
For some primary STS, involvement of vital organs or structures limits the potential for curative treatment. Distant metastasis may still be the commonest cause of death, but in a significant minority uncontrolled local tumour may be fatal. In the head and neck, retroperitoneum, paraspinal regions and visceral sites (bowel, uterus) tumours are often locally advanced at presentation, and/or locoregional relapse occurs frequently. The prognosis of primary tumours arising in such vital organs as the heart and great vessels, lung, liver, brain and spinal cord is even more ominous and most are ultimately fatal.

*Recurrence after surgery and radical radiotherapy*
Many of the tumours (e.g. retroperitoneal, head and neck, etc.) mentioned in the preceding section eventually fall into this category, as well as extremity sarcomas recurring in amputation sites. Curative options are limited for radiation-induced sarcomas in central locations. Desmoid tumours (aggressive fibromatosis), although they do not metastasise, have a high rate of recurrence that may be debilitating and cause the death of a small minority of patients.[1]

*(2) Presence of lymph node metastases*
Although rare in adult STS, lymph node metastases denote advanced disease. As well as being a marker of poor prognosis, categorised as stage IV disease by UICC staging,[2] their presence particularly at geographically separate sites complicates local management.

### *Distant metastases*

In common with many cancers, STS can disseminate widely, and most patients with distant metastases have incurable disease. Evidence for the contention that, for some patients, aggressive treatments can eradicate metastatic sarcoma, prolong life and cure a minority of patients will be examined.

Correspondence to: Vivien Bramwell, Department of Medicine, Tom Baker Cancer Centre, 1331 29th Street N.W., Calgary, Alberta, T2N 4N2, Canada. E-mail: vivienbr@cancerboard.ab.ca

ISSN 1357-714X print/1369–1643 © 2003 Taylor & Francis Ltd
DOI: 10.1080/13577140310001607266

### Epidemiology of advanced sarcoma

*Presentation 'de novo' with advanced STS*

Based on data from the National Cancer Institute Surveillance, Epidemiology and End Results (SEER) database 1986–1990, the estimated annual incidence of adult STS (excluding Kaposi sarcoma) is around four to five per 100 000 population, which represents less than 1% of all malignancies.[3]

Statistics on the incidence of metastases at presentation of STS, and subsequent local and regional failure rates are provided in reports from a large American cancer registry. Lawrence *et al.*[4] described results of a Pattern of Care Survey by the Committee on Cancer of the American College of Surgeons (CCACS) for the years 1977–1978 and 1983–1984. This CCACS database was expanded to become a joint project with the American Cancer Society, termed the National Cancer Data Base (NCDB), and Pollak *et al.*[5] reported updated information in 1996. Approximately 23% of adults with STS had metastases at presentation and this varied from 18 to 35% by primary site (Table 1). Lung was the most frequent single site, but only represented a third of metastatic lesions (Table 2).

*Development of advanced STS after primary treatment*

Data from the CCACS and NCDB reports, on patients presenting without metastases and followed for more than 5 years, showed a locoregional failure rate of around 19%, and distant failure in 18–20% of cases, which also varied by primary site (Table 3).

The CCACS and NCDB databases are retrospective and depend on acute care hospitals voluntarily reporting cases to a computerised cancer registry. Central pathology review was not performed and staging was a composite of clinical and pathological stages. Data on incidence and sites of recurrence were missing for many cases, limiting the reliability of conclusions based on a proportion of patients. Although results from the databases of single institutions may be distorted by referral patterns, they can provide in depth data on outcomes.

Pisters *et al.*[6] used a prospectively established database at Memorial Sloan Kettering Cancer

**Table 1.** *Presentation with metastatic soft tissue sarcoma*

| Study (Reference) | Year | Number of patients | Sites | % Metastases |
|---|---|---|---|---|
| CCACS (4) | 1977–78 | 2355 | All | 23.4 |
| | 1983–84 | 3457 | All | 23.3 |
| NCDB (5) | 1988 | 3500 | All | 20.5 |
| | 1983 | 4252 | All | 20.9 |
| | | 940 | Heart, mediastinum, pleura | 35 |
| | | 108 | Peripheral/autonomic nervous system | 21 |
| | | 528 | Peritoneum/retroperitoneum | 35 |
| | | 2676 | Connective/subcutaneous/other soft tissue | 18 |

**Table 2.** *Sites of metastases* of soft tissue sarcoma*

| | | % Patients by metastatic site | | | |
|---|---|---|---|---|---|
| Study (Reference) | Year | Lung | Bone | Liver | Other |
| CCACS (4) | 1977–78 | 33 | 23 | 15 | 26 |
| | 1983–84 | 34 | 24 | 16 | 24 |

*Data not available in NCDB report.

**Table 3.** *Treatment failure of soft tissue sarcoma*

| | | | | % Recurrence | |
|---|---|---|---|---|---|
| Study (Reference) | Year | Number of patients | Sites | Locoregional | Metastases |
| CCACS (4) | Not stated | 1209* | All | 19.5 | 17.9 |
| NCDB (5) | 1988 | 833* | All | 18.8 | 19.7 |
| | | | Heart, mediastinum, pleura | | 8.2 |
| | | | Peripheral/autonomic nervous system | | 28.6 |
| | | | Peritoneum/retroperitoneum | | 12.3 |
| | | | Connective tissue/subcutaneous/ other soft tissue | | 22.9 |

*Subset patients with adequate data.

Centre (MSK) to analyse prognostic factors and outcome for 1041 patients with localised STS of the extremities. The MSK database spanned 1982–1994, and the median follow-up time for all patients was 3.95 years. (The MSK database has been used to analyse many different questions relating to STS. For several reports quoted in this chapter, the years spanned in the analyses and patient numbers differ between studies, but all are using a similar data set). Although 181 patients (17%) developed local recurrence, in the absence of metastases most could be salvaged by further locoregional treatment. Two hundred and twenty-four patients (22%) developed distant metastases within a median time of 13 months. Sixty-eight had distant metastasis that occurred synchronously with, or subsequent to, a local recurrence. In the Cox multivariate analysis, relative risk (RR) of distant recurrence was greater for large tumour size (5–10 cm, RR 1.9; >10 cm, RR 1.5), presentation with locally recurrent disease (RR 1.5), deep tumour location (RR 2.5) and high grade (RR 2.5). With other prognostic factors balanced in the regression analysis, histological subtypes leiomyosarcoma (RR 1.7) and liposarcoma (RR 0.64) were adverse and favourable prognostic factors, respectively.

The French Federation of Cancer Centres[7] has established a cooperative database with collegial pathology review. Between 1980 and 1994, 1240 patients with localised STS were included, not only extremity STS (731 patients) but 80 head and neck sarcomas, 232 of the trunk wall and 182 internal trunk cases (retroperitoneum, abdominal cavity and pelvis). By multivariate analysis, unfavourable characteristics predicting the development of distant metastases were high grade (RR 7.8), size >10 cm (RR 2.02) bone or neurovascular invasion (1.5) and deep location (1.47).

### Clinical features/evolution of advanced sarcoma

*Prognostic factors/outcome for patients with advanced STS*

*Localised sarcomas at specific sites*

STS occurring at certain sites are highly likely to be advanced at presentation or to progress locally (with or without metastases) because of anatomic constraints on treatment. These problems are reflected in 5-year survival data by site from the NCDB database: 14.3% for heart/mediastinum/pleura; 67.1% for peripheral/autonomic nervous system; 46.1% for peritoneum/retroperitoneum; and 67.4% for connective/subcutaneous/other soft tissue locations.

There is increasing evidence that mesenchymal tumours of the gastrointestinal tract, previously labelled leiomyosarcomas, are a distinct clinicopathological entity.[8] Now classified as gastrointestinal stromal tumours (GIST) they are thought to arise from the interstitital cell of Cajal, an intestinal pacemarker cell.[9] These cells exhibit both smooth muscle and neural differentiation, and express the hematopoietic progenitor cell marker CD34, as well as the *c-kit* tyrosine kinase.[10] These tumours have a high recurrence rate.[11] Ng *et al.* found that only 13/132 (10%) of patients with initial complete resection were free of disease at a median follow-up of 68 months. Factors significantly associated with improved survival after relapse were initial disease-free interval of $\geq$18 months, recurrences either isolated to the peritoneal cavity or within the liver, or complete resection of peritoneal recurrence or liver metastases.[12] Similarly, with a median follow-up of 24 months (range 1–175), DeMatteo *et al.*[13] reported recurrences in 32 (40%) of 80 patients undergoing complete resection of GIST, with a 5-year disease-specific survival of 54%. Survival was predicted by tumour size but not microscopic margins of resection.

*Lymph node metastasis*

The MSK database[14] has also been used to examine the prevalence and natural history of lymph node metastasis in adult STS. Of 1772 patients with STS at all sites registered between 1982 and 1991, 46 (2.6%) showed lymph node metastases. Two large literature reviews documented a higher incidence: 9.1%[15] and 10.8%,[16] but may have suffered from a reporting bias. In the MSK data set, tumour types with the highest prevalence of lymph node metastases were angiosarcoma 5/37 (13.5%), embryonal rhabdomyosarcoma 12/88 (13.6%) and epithelioid sarcoma 2/12 (16.7%). Although most of the remaining cases were in leiomyosarcoma and malignant fibrous histiocytoma (MFH), this only represented incidence rates of 2.5 and 2.6% in each of these subtypes. In all but one case, the primary tumours were high grade sarcomas. Median survival from the time of primary diagnosis was 30 months and from the time of lymph node metastasis was 12.8 months. By univariate analysis, visceral location of the primary, histological type MFH and limited surgery for lymph node metastases were found to confer a poor prognosis. These findings were based on small numbers with short follow-up and should be interpreted with caution.

*Distant metastases*

The 5-year survival data for patients with stage IV disease at presentation was similar for both CCACS and NCDB studies, around 19%. The CCACS study also analysed the results of salvage therapy by site of recurrence, reporting 5-year survival rates of 60.5, 20.8 and 9.8% for patients with local relapse only, patients with lung metastases only and patients with multiple metastatic sites.

Billingsley *et al.*[17] used the MSK database 1992–1996, comprising 994 adult patients with primary extremity STS, to analyse survival of patients with distant metastasis. The median follow-up was 33 months, during which 230 (23%) patients developed metastases. The lungs were the first site of metastasis in 169 patients (73%), with soft tissues (10%) being the next most common site. The median survival after diagnosis of metastases was 11.6 months, and actuarial survival at 2 years was 28% (median follow-up 10 months). By multivariate analysis, adverse prognostic factors for post metastasis survival were unresectable metastatic disease (RR 2.3; $P=0.0001$), local recurrence with or before distant metastasis, (RR 2.0; $P=0.01$), disease-free interval <1 year (RR 1.4; $P=0.03$) and age >50 years (RR 1.4; $P=0.05$). Other factors such as metastatic disease limited to one lung and characteristics of the primary tumour had no significant effect on outcome after first metastasis.

*Common clinical problems/causes and modes of death*

*Locoregional STS*

Given the diversity of possible primary sites, locally advanced incurable STS can present with a large range of symptoms and signs. Most of these are the consequence of a locally expanding mass causing pressure on, or destruction of, adjacent tissues. This damage to soft tissues may cause pain, ulceration and bleeding; in bone/joints it can lead to pain, fracture, joint effusion, loss of function; and in nerves/spinal cord result in pain and loss of function (numbness or muscle weakness). Within the body cavities a variety of effects may be seen, such as bleeding, perforation or obstruction of the gastrointestinal and genitourinary tracts; and effusions (pleural, pericardial), bleeding and respiratory obstruction caused by intrathoracic STS. Causes of death associated with uncontrolled locoregional sarcoma usually relate to catastrophic bleeding, infection, obstruction (most commonly bowel or renal) and thromboembolic disease.

*Distant metastases*

The lungs and pleura are the commonest sites of metastasis from STS. Symptoms may not appear until lung metastases reach a substantial size and/or number. Among the other factors determining the appearance of symptoms are patient activity level, pulmonary reserve, and location of the metastases. Effusions commonly develop in conjunction with pleural based metastases. Death is usually due to respiratory failure and/or infection.

Retroperitoneal and visceral (gastrointestinal, genitourinary) STS often metastasise to the liver, and hepatic metastasis is occasionally seen from other primary sites. Early symptoms are nausea, fatigue, satiety followed by pain, abdominal swelling and jaundice, ultimately leading to hepatic failure. GIST, and some other intra-abdominal retroperitoneal tumours, may disseminate widely within the abdomen. Patients with extensive intra-abdominal disease may be remarkably free of symptoms for long periods. Eventually patients succumb to subacute/acute obstruction or perforation/bleeding.

Bone metastases occur infrequently, being less commonly associated with STS than with bone sarcomas. Pain and fractures are the commonest complications but skeletal metastases are rarely a direct cause of death. Central nervous system metastases are rare, but brain metastases may present with headaches or central neurological deficits. Spinal cord compression may be seen from epidural metastases, collapse of vertebrae due to bone metastases or locoregional invasion by STS.

Symptomatic and supportive care measures used to deal with complications of uncontrolled local or metastatic tumours, vary by site and are beyond the scope of this article.

### Treatment of soft tissue sarcomas

*Surgery*

*Debulking of primary tumour*

Partial removal (debulking) of locally recurrent STS is rarely beneficial, especially if the recurrence is in an irradiated field. There is a risk of rapid progression/local recurrence in the operative site. A rare exception might be made for a slowly evolving low grade STS, especially within the abdominal cavity, or borderline tumours such as fibromatoses. Debulking associated with radiotherapy and/or chemotherapy is discussed below.

*Resection of metastases*

*Pulmonary.* It is generally accepted that, in selected cases, pulmonary metastatectomy is potentially curative, although this has never been confirmed in a RCT. Most patients with isolated pulmonary metastases can be considered for metastatectomy. In a review of the English language literature 1978–1994, comprising 12 case series totalling 697 patients, Frost[18] identified three pretreatment adverse prognostic factors: (1) tumour doubling time <40 days; (2) >4 nodules; (3) disease-free interval <12 months. Incomplete resection is associated with a poor outcome. Patients with mediastinal lymphadenopathy and/or tumour-related pleural effusion should not be considered. Frost found the 5-year survival rates to range from 15 to 35% for first time pulmonary metastatectomy and from 12 to 52% for reoperations, with a median value of 25% in all patients undergoing resection.

*Other sites.* Therapeutic lymphadenectomy with curative intent was performed in 31 of 46 cases of

STS with lymph node metastases registered in the MSK database described earlier.[14] Median survival for these patients was 16.3 months and 46% survived 5 years. The 15 patients not treated by lymphadenectomy did poorly with a median survival of 4.3 months (range 1–32). This is a biased comparison, as fitter patients may have been selected for surgery. If technically feasible, the primary tumour is controlled and there are no distant metastases, radical lymphadenectomy is likely to produce good palliation with the potential for cure.

Investigators from MSK have reported their experience of liver resection in 96 patients with hepatic metastases from non-colorectal, non-neuroendocrine cancers, 41 of whom had STS.[19] Median survival after hepatic resection of STS was 31 months, and there was one 5-year survivor. Disease-free interval >36 months before detection of liver metastases, complete resection and primary tumour group (genitourinary cancers > STS > gastrointestinal cancers) were predictors of a significantly better survival, by multivariate analysis.

In a prospective protocol of debulking surgery, Karakousis *et al.*[20] included 72 consecutive patients with STS disseminated within the abdomen. Median survival from first exploratory surgery was 23 months for the 46 patients (64%) in whom complete resection was possible. Median survival times for grade I, II, II tumours were 35.4, 17.5, 14.5 months, respectively ($P < 0.01$) and for patients undergoing complete resection, medial survival was better for completely resected cases with a disease-free interval >36 months.

The incidence of brain metastases from STS is low, and varies with histology from 1 to 8%. Incidence is increasing because, it is suggested, of prolonged survival associated with improved systemic control of disease. Further, many chemotherapeutic agents fail to cross the blood–brain barrier.[21] In a case series of 21 patients with brain metastases from a variety of bone and soft tissue sarcomas,[22] median survival after craniotomy was 11.8 months. No patient survived 5 years, but six were alive at the time of reporting, the longest surviving 25 months. As for lung and liver metastases, complete removal is critical for long-term survival.

### *Radiotherapy*

### *Alternative radiotherapy techniques for locoregional recurrence*

Most patients who develop inoperable local recurrence have previously received radical radiation to doses in excess of 6000 cG (or lower doses within the abdomen but still close to tolerance of normal tissue). Those who have not should be considered for radical locoregional radiotherapy which, even in the presence of bulky sarcoma, may produce long-term control.[23] Additional palliative irradiation may be possible at previously irradiated sites to relieve symptoms such as pain, bleeding, loss of function, etc. The benefits of treatment versus the long-term radiation complications must be assessed in the context of the life expectancy of the patient.

Brachytherapy has been successful in controlling STS recurrence after previous surgery and external beam radiation (EBR), although most of the reported experience relates to extremity sarcoma.[24] High linear energy transfer (LET) radiation therapy such as neutrons produces high rates of local control in patients with macroscopic residual STS and unresectable tumours <10 cm,[25] but at a cost of substantial toxicity. Increasing use of conformal therapy and light ion beam therapy, combining the dose distribution advantages of protons with the biological properties of high LET particles,[26] may improve results. Intra-operative irradiation, which permits delivery of a large radiation dose directly to the tumour mass while sparing normal tissue such as bowel, has produced promising results in retroperitoneal STS[27] when used in combination with surgery and EBR. Data in this area are sparse, and as current fiscal realities limit availability of these costly machines and facilities, treatment should be given only in a trial setting.

### *Radiotherapy for metastases*

For STS metastases at many sites, EBR may be an effective palliative treatment. It should be reserved for symptomatic disease, or involvement of sites likely to cause severe complications, such as incipient spinal cord compression or risk of pathological fracture. Symptoms like pain, loss of function, bleeding, obstruction may be relieved by appropriately focussed EBR. The modality is particularly useful for metastases in bone, soft tissue, paraspinal and pelvic regions. EBR is of little value in common sites of STS dissemination, such as multiple lung or liver metastases, pleural effusion or widespread intraperitoneal disease.

### *Chemotherapy*

### *Standard dose chemotherapy*

It is generally accepted that the anthracyclines (doxorubicin (DOX), epirubicin (EPI) and ifosfamide (IFOS)) are the most active single agents in adult STS,[28,29] with single agent response rates in the range 20–30%. Dacarbazine (DTIC) also has limited activity. Although marginal activity in the 10–15% range has been documented for a large number of other agents and some have been incorporated in combination regimens, it is doubtful whether they contribute anything other than toxicity. Etoposide is said to be synergistic with IFOS and this is a well-established combination regimen in paediatric sarcomas.[30] Etoposide is inactive in adult

STS[31,32] and it is also unclear from published pilot studies[33,34] whether the combination is more active than IFOS alone.

Despite the extensive literature on a variety of combination chemotherapy regimens, it is still difficult to establish the most effective systemic treatment for advanced STS. Indeed, it can be questioned whether combination chemotherapy has any advantages over the sequential use of active single agents. A meta-analysis[35] of eight RCTs comparing single agent DOX with 10 DOX-based combination regimens in 2281 patients showed only a non-significant trend for improved response rate with combination chemotherapy (OR = 0.78, 95% CI 0.60–1.05, $P = 0.10$) and no benefit for overall survival (OR = 0.84, 95% CI 0.67–1.06, $P = 0.13$). Considering only the two RCTs that included combination regimens using optimal standard doses of DOX and IFOS, an ECOG (Eastern Cooperative Oncology Group) study[36] showed a higher response rate of 34% for DOX/IFOS compared with 20% for DOX alone ($P = 0.03$), whereas in an EORTC study[37] respective response rates were 23 and 28% for DOX and DOX/IFOS ($P$ not significant). In neither study was overall survival different between the arms. Although the addition of IFOS to DOX/DTIC increased the response rate (32 vs. 17%, $P < 0.002$) in an Intergroup RCT,[38] it had no impact on survival.

In conclusion, if palliation of symptomatic metastatic disease is the goal of therapy, this is likely to be best achieved by sequential single agent therapy.

### High dose chemotherapy

Historically myelosuppression, particularly neutropenia with the risk of infection, has been dose limiting for many chemotherapy agents and combinations. The widespread availability of hemopoietic growth factors (granulocyte or granulocyte/macrophage colony stimulating factors (G-CSF, GM-CSF)) permits the exploration of high-dose chemotherapy. This topic has recently been reviewed.[39]

*(a) Dose escalation of individual active agents.* Dose escalation of DOX and its analogues continues to be limited by cardiotoxicity, despite the introduction of dexrazoxane.[40,41] Liposomal encapsulation of anthracyclines may alter the spectrum of toxicity, but their benefit in terms of prevention of cardiotoxicity remains to be proven. In an EORTC (European Organization for Research and Treatment of Cancer) RCT[42] liposomal doxorubicin had equivalent efficacy to DOX, with a lower incidence of febrile neutropenia, but more skin toxicity and hypersensitivity. Whether such a toxicity profile will permit dose escalation is uncertain at present.

In STS most attention has been paid to dose escalation of IFOS. Doses of 12 $g/m^2$ without, and 14–18 $g/m^2$ with growth factor support, seem achievable, but are often associated with high incidences of nephro- and neurotoxicity. In these exploratory studies, often conducted in patients who have received previous chemotherapy that might have included standard dose IFOS, response rates have varied substantially from 0 to 46%.[43–47] Differences in patient populations between studies probably account for the discrepancies. Whether intravenous intermittent daily bolus or continuous infusion is the better schedule[44,49] has not been resolved, although a recent EORTC study found no difference in response rates, progression-free and overall survival between these two methods of administration of IFOS 9 $g/m^2$.[50] Prolonged infusion over 21 days may be a less toxic way to administer high dose IFOS.[51]

*(b) Dose escalated combination chemotherapy.* Several groups have conducted phase I and II studies of high-dose anthracycline/IFOS combinations with or without DTIC, with response rates ranging from 31 to 67%.[52–64] Toxicities have been severe, particularly thrombocytopenia, and the neuro- and nephrotoxicities of IFOS. At these doses neutropenic fevers are common, for growth factors do not completely protect against myelosuppression.

Preliminary results on response rates in two RCTs, evaluating moderate dose escalation supported by G-CSF, are not encouraging. A RCT[65] comparing standard dose DOX/IFOS (previously used by EORTC) with the same regimen with a 50% dose escalation of DOX, showed similar response rates, 20 vs. 21% in 314 patients. Bui *et al.*[66] compared standard dose MAID with the same regimen dose escalated 25%, and showed respective response rates of 37% in 76 patients and 43% in 72 patients (not significantly different). Doses of DOX (75 $mg/m^2$) and IFOS (5 $g/m^2$) administered in these studies were lower than in many of the high-dose phase I and II studies, but such dose-intensified treatments may only be tolerated by a select patient population (age <65, XRT to <20% marrow, performance status 0–1, no prior chemotherapy) as acknowledged by Patel *et al.*[58] This topic is reviewed in a series of papers "Should high-dose chemotherapy be used in the treatment of soft tissue sarcoma?" providing pro, contra and arbiter views.[67]

*(c) High-dose chemotherapy with autologous marrow (ABM) or stem cell (SC) support.* Data on very high-dose chemotherapy with ABM/SC support are even more sparse.[68–79] Although these studies document the feasibility of a variety of high-dose protocols, the small numbers of patients in each precludes any accurate assessment of benefits. Negative results for high-dose chemotherapy and ABM/SC in two large RCTs[80,81] in metastatic breast cancer do not augur well for success in metastatic STS.

In conclusion, high-dose regimens should be evaluated against standard treatment in RCT that include quality of life and economic endpoints. Currently their use for disease palliation outside a clinical trial setting is not recommended. Their potential value as adjuvant treatment, or for the aggressive management of young patients with metastatic disease merits further exploration.

*Novel/investigational treatments*

Expanding knowledge of both cancer cell biology and the process of metastasis has led to the development of a range of novel compounds. Drugs that interrupt cell signalling pathways,[82] modulate drug resistance mechanisms, or interfere with malignant cell invasion (matrix metalloproteinase inhibitors) and/or angiogenesis,[83] are now available. Specific vaccines and immune modulators are under development.[84]

For example, troglitazone activates the ligand for the PPAR$\gamma$ nuclear receptor and stimulates terminal differentiation in pre-adipocytes.[85,86] Demetri *et al.*[87] reported preliminary results of a phase II trial of this drug in 34 patients with liposarcoma. Biopsy samples were taken before and after treatment. Five of seven patients with biopsy evaluable myxoid/round cell liposarcoma exhibited lineage appropriate differentiation of the liposarcoma cells.

STS show high primary drug resistance. Poor efficacy/toxicity ratios may account for negative RCTs evaluating amphotericin B[88] and amiodarone.[89] The reduced toxicity of a new generation of compounds allows them to be tested at an appropriate dose. Preliminary results of the drug Biricodar (VX-710) that reverses two important mechanisms of resistance, MDR and MRP, are promising. Added to DOX, Biricodar induced 2PR in 15 non-GIST STS proven to be resistant to DOX alone.[90] Mechanisms of resistance for a variety of chemotherapy agents used in the treatment of STS are reviewed by Colvin *et al.*[91] STS are often characterised by acquired changes which affect G1 checkpoint control (e.g. Cdk over-expression) resulting in unregulated progression through the cell cycle. This provides the rationale for a Canadian Sarcoma Group study of flavopiridol, an agent that has inhibitory effects on several cyclin-dependent kinases.[92] Ecteinascidin 743 (ET-743), a novel minor groove DNA-binding agent specific to guanine–cytosine-rich regions, is showing promising activity in early phase II studies in STS.[93–95]

The most exciting development in systemic treatment for mesenchymal tumours is the striking activity of STI-571 in advanced and metastatic GIST. STI-571 is a rationally designed drug which selectively inhibits BCR-ABL, KIT and PDGFR tyrosine kinases, and has established activity in chronic myeloid leukaemia. GIST are characterised by expression of the proto-oncogene *c-kit* and contain gain of function mutations leading to ligand-independent activation. In European[96] and US[97] phase II studies, a majority of patients with GIST (who are notoriously resistant to conventional chemotherapy) have responded to daily oral doses of 600–800 mg of STI-571. Preliminary data from a randomised phase III trial (Intergroup S0033) evaluating two dose levels (400 vs. 800 mg/day) in patients with unresectable or metastatic GIST[98] documents response rates of 43 and 41%, respectively, with no differences in progression-free (80 and 82%) and overall survival (91 and 92%) at 6 months. It is anticipated that final response rates will be higher.

Chemotherapy may occasionally be indicated for desmoid tumours causing major symptoms and/or invading vital structures. However, Ballo *et al.*[1] reported 5-, 10- and 15-year survival rates of 96, 92 and 87%, respectively, for patients treated by surgical resection with or without radiotherapy, and Mitchell *et al.*[99] make a convincing case that these tumours can have prolonged periods of stable disease. This characteristic makes interpretation of response to treatment difficult. There are data from several sources documenting responses to low-dose, well-tolerated chemotherapy comprising weekly vinblastine and methotrexate,[100–102] and more aggressive chemotherapy of the type used for STS[103,104] should rarely be necessary unless low-dose chemotherapy has failed and/or disease threatens life or major organ function. Using immunohistochemistry and qualitative real-time polymerase chain reaction analysis, Mace *et al.*[105] demonstrated consistent positivity for KIT and PDGFR $\alpha$ and $\beta$ in nine desmoid tumour specimens. Two patients were treated with imatinib and demonstrated clinical and radiological responses ongoing at 9 and 11 months. An additional case report[106] documents a response to imatinib in dermatofibrosarcoma protuberans. Clearly further study is warranted in this difficult disease.

*Multimodality treatment*

An argument in favour of high-dose combination chemotherapy intended to maximise response is that combined with aggressive surgery it may lead to cure of metastatic STS. Small prospective studies have been performed,[107,108] and in some patients there has been long-term control of disease. However, metastatic STS can have an extremely variable natural history, and in the absence of appropriate randomised control groups and long-term follow-up it is difficult to determine overall benefit.

An interesting retrospective analysis of 38 patients achieving complete CR in Scandinavian Sarcoma Group studies[109] showed that those achieving CR by chemotherapy alone had a longer median survival

(23 months) than those who were converted to CR by surgery following chemotherapy (10 months). A good histological response to chemotherapy (defined in this study as no or few small areas of viable tumour) predicted a good outcome in patients subjected to surgery. It is conceivable that intrinsic drug sensitivity, rather than specific regimen or dose, is the main determinant of a good outcome for patients receiving chemotherapy.

Innovative routes for the delivery of chemotherapy in combined modality therapy include isolated lung perfusion for unresectable lung metastases,[110] hepatic chemoembolisation[111] and intraperitoneal treatment.[112,113] These procedures require a high level of technical expertise and should be done only in the context of a prospective clinical trial.

### *Factors predicting benefit of chemotherapy*

The most reliable data on prognostic factors for patients receiving chemotherapy for metastatic STS is from an analysis of an EORTC database, comprising 2185 patients receiving first line anthracycline-based chemotherapy in seven RCT spanning a period of 20 years.[114] Overall survival time (median 51 weeks) and response to chemotherapy (26% CR + PR) were used as the two major endpoints for a prognostic factor analysis. By multivariate analysis (Cox model) good performance status ($P < 0.0001$), absence of liver metastases ($P = 0.001$), low histopathological grade ($P = 0.0004$) and young age ($P = 0.0045$) were favourable factors for survival. Absence of liver metastases ($P < 0.0001$), young age ($P = 0.0024$), high histopathological grade ($P = 0.0051$) and liposarcoma ($P = 0.0065$) were favourable factors for response. By univariate analysis, synovial sarcoma subtype predicted a favourable response. However, this subtype was strongly correlated with young age. This may account for anecdotal information from Rosen's group that synovial sarcomas respond particularly well to IFOS,[115] as the age range in this study was 14–39 years. Blay *et al.*[116] analysed a subset of the same database, comprising 2187 patients receiving DOX chemotherapy in RCT between 1976 and 1990, and described features characterising long-term (5-year) survivors. There were 66 of 1888 patients alive at 5 years, who were more frequently: female (69 vs. 51%), had grade I tumours (35 vs. 11%), and had PS 0 (63 vs. 41%). Although CR on DOX was a major parameter correlated with 5-year survival, with 21% (17/81) being alive at 5 years, the fact that 17 of 323 patients (5%) with PR, 17 of 658 patients (3%) with SD and three of 630 (0.5%) with PD were also alive at 5 years illustrates the heterogeneity of outcome for patients with advanced STS. In a third analysis of the same database by Reichardt *et al*,[117] the presence of locally recurrent disease together with metastases predicted for a low response rate and poor survival.

### *Active treatment versus best supportive care: decision process*

For patients with non-resectable local or metastatic STS, the relative merits of active treatment with radiotherapy and/or chemotherapy versus symptomatic measures for individual patients may be assessed using the following criteria:

(a) Is there a remote chance of long-term control or cure with active treatment?
(b) Is the disease symptomatic, or are serious complications imminent?
(c) Is this the optimum time to intervene?
(d) Is it likely that the benefit/toxicity ratio of active treatment will be favourable (i.e. is treatment likely to improve quality of life)?
(e) How will co-morbidities (e.g. age, performance status, tumour burden, other illnesses) influence treatment outcomes?

### Summary of management

### *Locoregional disease*

Management of patients with inoperable local STS is challenging. Site of disease, the severity of symptoms, speed of tumour growth, age, performance status and comorbid diseases are factors that should be considered in formulating a treatment plan. This will be influenced by the preferences of patient and family. Long-term control is most likely to be achieved by a combination of chemotherapy with such local measures (surgery, radiotherapy) that are permitted by anatomic limitations and previous radical treatments. Speed of tumour growth is an important consideration — even advanced STS can be remarkably heterogeneous in this respect. For slow-growing STS, causing few symptoms, the best quality of life may be achieved by judicious observation and occasional symptomatic measures.

### *Metastatic disease*

Metastases at certain sites may be amenable to removal, with good palliative benefit and occasional cure. These sites may include lung, liver, abdominal cavity, lymph nodes and brain. Factors that, in general, will predict a favourable outcome include control of the primary tumour, one or small numbers of metastases in one site which are fully resectable, slow tumour growth (often indicated by a long disease-free interval from primary resection to development of metastases $\geq 2$ years) and good performance status/lack of comorbid diseases.

In patients with metastases that are completely resectable the role of chemotherapy remains controversial, and surgery alone is a reasonable first approach. Chemotherapy or, at selected sites, radiotherapy, may be added if resection is incomplete, but the benefit of such treatments is not established. Alternatively, if disease is surgically accessible, complete resection may prolong CR and PR achieved with chemotherapy. If combined modality treatment is planned, initial chemotherapy permits a determination of chemosensitivity.

Disease progression in patients with GIST can be highly variable, and it is worth following asymptomatic patients to determine the rate of growth of metastases before considering systemic treatment. Imatinib is now available for palliative treatment of patients with symptomatic progressive metastatic GIST, and is being tested in the adjuvant setting.

In contrast to ASTS, histological subtypes of sarcomas commonly seen in the paediatric age group (embryonal rhabdomyosarcomas, primitive neuroectodermal tumours) may also be chemosensitive in adult patients, and such patients should receive intensive multiagent chemotherapy. However, results for adult patients are generally poorer than for children. Esnaola *et al.*[118] reported outcomes of treatment for 39 adults, median age 26 (16–82) years, with rhabdomyosarcoma (embryonal, seven; alveolar, 22; pleomorphic, 10; not specified, five) treated at their institutions between 1973 and 1996. Twenty-six had locoregional disease and 13 metastatic disease at presentation. Thirty-seven patients received chemotherapy, with high overall (72%) and complete response (41%) rates. Nevertheless, 5- and 10-year overall survival rates were low, 31 and 27%, respectively. Patients with locoregional disease had a 44% 5-year survival rate, but there were no survivors among patients with metastatic disease. Data from another small series of juvenile-type STS in adults suggests that the long-term outlook is poorer than in children.[119]

For patients with metastatic STS the ultimate goals of chemotherapy will determine whether or not it should be given and if so, its timing and type. The exact role and benefits of chemotherapy has been a controversial subject over many years.[120–123] Young fit patients may be willing to risk substantial toxicity for a chance to maximise response to chemotherapy with the possibility of long-term control and a remote chance of cure. Early treatment with an aggressive high-dose combination chemotherapy regimen ± growth factors may be most appropriate for these patients. However, many patients with STS are elderly with other health problems, and palliation of symptoms is the main objective. In this situation, the ideal time to initiate chemotherapy is when the patient is starting to get symptoms or has disease likely to cause major complications leading to a deterioration in performance status. A reasonable option for these patients is sequential single agents, e.g, DOX followed by IFOS at the time of relapse. If there is clearly measurable disease at the time of initiation of chemotherapy it should be possible to determine response within two to three cycles and terminate chemotherapy if it is ineffective. Response is unlikely if there is clear progression of disease in the first two cycles, but stabilisation of disease after previous rapid progression is an indication for continued therapy.

Because of the high rate of intrinsic drug resistance, and the limited number of effective drugs, further salvage chemotherapy after first line failure of combination chemotherapy is rarely successful. For patients in relapse after previous response, higher-dose IFOS might be an option. If phase I or II trials are available locally, these are opportunities for patients actively seeking further treatment.

### Conclusions

There is much to offer patients with advanced STS in the form of specific anticancer and supportive therapies. Nevertheless, there is much to learn and a great need for well-designed studies, particularly large phase III RCTs addressing important questions. What are the questions? In the context of this chapter a non-exhaustive list would be:

1. What is the role of newer techniques/energies of radiotherapy in controlling inoperable, recurrent and/or metastatic STS?
2. Is surgical resection of metastases beneficial in STS, and might this vary with sites of metastases?
3. Is 'high-dose' better than 'standard-dose' chemotherapy? This would include investigation of the feasibility/safety of such treatments in different patient populations with STS.
4. Do the benefits of chemotherapy vary according to histological subtype?
5. Is it feasible to target chemotherapy to different histological subtypes/grades/other tumour characteristics according to mechanisms of drug action?
6. Does chemotherapy before or after surgery for metastases improve survival?

### References

1. Ballo MT, Zagars GK, Pollack A, Pisters WT, Pollock RA. Desmoid tumor: prognostic factors and outcome after surgery, radiation therapy or combined surgery and radiation therapy. *J Clin Oncol* 1999; 17: 158–67.
2. International Union Against Cancer. *TNM Classification of Malignant Tumors*. 5th edn. Sobin LH, Wittekind Ch, eds. 1997. New York: John Wiley.
3. Zahm SH, Tucker MA, Fraumeni JF. Soft tissue sarcomas. In: Schottenfeld D, Fraumeni JF, eds. *Cancer Epidemiology and Prevention*. 1996; 984–99.
4. Lawrence W, Donegan WL, Natarajan N, Mettlin C, Beart R, Winchester D. Adult soft tissue sarcomas: a pattern of care survey of the American College of Surgeons. *Ann Surg* 1987; 205: 349–59.
5. Pollock RE, Karnell LH, Menck HR, Winchester DP. The National Cancer Database report on soft tissue sarcoma. *Cancer* 1996; 78: 2247–57.

6. Pisters PWT, Leung DHY, Woodruff J, Shi W, Brennan MF. Analysis of prognostic factors in 1,041 patients with localized soft tissue sarcomas of the extremities. *J Clin Oncol* 1996; 14: 1679–89.
7. Coindre J-M, Terrier P, Guillou L, Le Doussal V, Collin F, Ranchère D, Sastre X, Vilain M-O, Bonichon F, Bui BN. Predictive value of grade for metastasis development in the main histologic types of adult soft tissue sarcomas. *Cancer* 2001; 91: 1914–26.
8. Miettinen M, Sarlomo-Rikala M, Lasota J. Gastrointestinal stromal tumors: Recent advances in understanding of their biology. *Hum Pathol* 1999; 30: 1213–20.
9. Chan JK. Mesenchymal tumors of the gastrointestinal tract: A paradise for acronyms (STUMP, GIST, GANT, and now GIPACT), implication of c-kit in genesis, and yet another of the many emerging roles of the interstitial cells of Cajal in the pathogenesis of gastrointestinal diseases. *Am J Pathol* 1998; 152: 1259–69.
10. Kindbloom LG, Remotti HE, Aldenberg F, Meiss-Kindbloom JM. Gastrointestinal pacemaker cell tumor (GIPACT) gastrointestinal stromal tumors show phenotypic characteristics of the interstitial cells of Cajal. *Adv Anat Pathol* 1999; 6: 19–40.
11. Somerhausen NDSA, Fletcher CDM. Gastrointestinal stromal tumours: an update. *Sarcoma* 1998; 2: 133–41.
12. Ng E-H, Pollock RE, Romsdahl MM. Prognostic implications of patterns of failure for gastrointestinal leiomyosarcomas. *Cancer* 1992; 69: 1334–41.
13. DeMatteo RP, Lewis JJ, Leung D, *et al.* Two hundred gastrointestinal stromal tumors: Recurrence patterns and prognostic factors for survival. *Ann Surg* 2000; 231: 51–8.
14. Fong Y, Goit DG, Woodruff JM, Brennan MF. Lymph node metastasis from soft tissue sarcoma in adults: analysis of data from a prospective database of 1772 sarcoma patients. *Ann Surg* 1993; 217: 72–7.
15. Weingrad DN, Rosenberg SA. Early lymphatic spread of osteogenic and soft tissue sarcomas. *Surgery* 1978; 84: 231–40.
16. Mazeron JJ, Suit HD. Lymph nodes as sites of metastases from sarcomas of soft tissue. *Cancer* 1987; 60: 1800–8.
17. Billingsley KG, Lewis JJ, Leung DHY, Casper ES, Woodruff JM, Brennan MF. Multifactorial analysis of the survival of patients with distant metastasis arising from primary extremity sarcoma. *Cancer* 1999; 85: 389–95.
18. Frost DB. Pulmonary metastasectomy for soft tissue sarcomas: Is it justified? *J Surg Oncol* 1995; 59: 110–5.
19. Harrison LE, Brennan MF, Newman E, Fortner JG, Picardo A, Blumgart LH, Fong Y. Hepatic resection for noncolorectal, nonneuroendocrine metastases: a fifteen-year experience with ninety-six patients. *Surgery* 1997; 121: 625–32.
20. Karakousis CP, Blumenson LE, Canavese G, Rao U. Surgery for disseminated abdominal sarcoma. *Am J Surg* 1992; 163: 560–4.
21. Espana P, Chang P, Wiernik PH. Increased incidence of brain metastases in sarcoma patients. *Cancer* 1980; 45: 377–80.
22. Bindal RK, Sawaya RE, Leavens ME, Taylor SH, Guinee VF. Sarcoma metastatic to the brain: Results of surgical treatment. *Neurosurgery* 1994; 35: 185–91.
23. Slater JD, McNeese MD, Peters LP. Radiation therapy for unresectable soft tissue sarcomas. *Int J Rad Oncol Biol Phys* 1986; 12: 1729–34.
24. Nori D, Shupak K, Shiu MH, Brennan MF. Role of brachytherapy in recurrent extemity sarcoma in patients treated with prior surgery and irradiation. *Int J Radiat Oncol Biol Phys* 1991; 20: 1229–33.
25. Stannard CE, Vernimmen FJ, Jones DTL, Van Wijk AL, Brennan SM, Visser AM, *et al.* The neutron therapy clinical programme at the National Accelerator Centre (NAC). *Bull Cancer/Radiotherapy* 1996; 83: 87–92s.
26. Chauvel P. Osteosarcomas and adult soft tissue sarcomas: is there a place for high LET radiation therapy? *Ann Oncol* 1992; 3: S107–10.
27. Gunderson LL, Nagorney DM, McIlrath DC, Fieck JM, Wieand HS, Martinez A, *et al.* External beam and intraoperative electron irradiation for locally advanced soft tissue sarcomas. *Int J Radiat Oncol Biol Phys* 1993; 25: 647–56.
28. Demetri GD, Elias AD. Results of single-agent and combination chemotherapy for advanced soft tissue sarcomas: implications for decision making in the clinic. *Hematol Oncol Clin N Am* 1995; 9: 765–85.
29. Keohan ML, Taub RN. Chemotherapy for advanced sarcoma: therapeutic decisions and modalities. *Sem Oncol* 1997; 24: 572–9.
30. Miser JS, Kinsella TJ, Triche TJ, Tsokos M, Jarosinski P, Forquer R, *et al.* Ifosfamide with mesna uroprotection and etoposide: an effective regimen in the treatment of recurrent sarcomas and other tumors of children and young adults. *J Clin Oncol* 1987; 5: 1191–8.
31. Keizer HJ, Crowther D, Nielsen OS, Van Oosterom AT, Muguiro HJ, Van Pottelberghe C, *et al.* EORTC group phase II study of oral etoposide for pretreated soft tissue sarcoma. *Sarcoma* 1997; 1: 99–101.
32. Crawley CR, Judson IR, Verrill M, Hill C, Raynaud FI. A phase I/II study of a 72-h continuous infusion of etoposide in advanced soft tissue sarcoma. *Sarcoma* 1997; 1: 149–54.
33. Edmonson JH, Buckner JC, Long HJ, Loprinzi CL, Schaid DJ. Phase II study of ifosfamide-etoposide-mesna in adults with advanced nonosseous sarcomas. *J Natl Cancer Inst* 1989; 81: 863–6.
34. Saeter G, Alvegard TA, Monge OR, Strander H, Turesson I, Klepp R, *et al.* Ifosfamide and continuous infusion etoposide in advanced adult soft tissue sarcoma. A Scandinavian Sarcoma Group phase II study. *Eur J Cancer* 1997; 33: 1551–8.
35. Bramwell VHC, Anderson D, Charette M. Doxorubicin-based chemotherapy for the palliative treatment of adult patients with locally advanced or metastatic soft tissue sarcoma – a meta-analysis and clinical practice guideline. *Sarcoma* 2000; 4: 103–12.
36. Edmonson JH, Ryan LM, Blum RH, Brooks JSJ, Shiraki M, Frytak S, Parkinson DR. Randomized comparison of doxorubicin alone versus ifosfamide plus doxorubicin or mitomycin, doxorubicin, and cisplatin against advanced soft tissue sarcomas. *J Clin Oncol* 1993; 11: 1269–75.
37. Santoro A, Tursz T, Mouridsen H, Verweij J, Steward W, Somers R, *et al.* Doxorubicin versus CYVADIC versus doxorubicin plus ifosfamide in first-line treatment of advanced soft tissue sarcomas: a randomized study of the European Organization for Research and Treatment of Cancer Soft Tissue and Bone Sarcoma Group. *J Clin Oncol* 1995; 13: 1537–45.
38. Antman K, Crowley J, Balcerzak SP, Rivkin SE, Weiss GR, Elias A, *et al.* An Intergroup phase III randomized study of doxorubicin and dacarbazine with or without ifosfamide and mesna in advanced soft tissue and bone sarcomas. *J Clin Oncol* 1993; 11: 1276–85.

39. Verma S, Bramwell V. Dose-intensive chemotherapy in advanced adult soft tissue sarcoma. *Expert Rev Anticancer Ther* 2002; 2: 7201–15.
40. Lopez M, Vici P, Di Lauro L, Conti F, Paoletti G, Ferraironi A, *et al.* Randomized prospective clinical trial of high-dose epirubicin and dexrazoxane in patients with advanced breast cancer and soft tissue sarcomas. *J Clin Oncol* 1998; 16: 86–92.
41. Seymour L, Bramwell V, Moran LA, *et al.* Use of dexrazoxane as a cardioprotectant in patients receiving doxorubicin or epirubicin chemotherapy for the treatment of cancer. *Cancer Prev Control* 1999; 3: 145–59.
42. Judson I, Radford J, Harris M, Blay J-Y, van Hoesel Q, le Cesne A, *et al.* Randomized phase II trial of liposomal doxorubicin (Doxil/Caelyxl) versus doxorubicin in the treatment of advanced or metastatic soft tissue sarcomas – a study by the EORTC Soft Tissue and Bone Sarcoma Group trial. *Eur J Cancer* 1999; 37; 870–7.
43. Le Cesne A, Antoine E, Spielmann M, Le Chevalier T, Brain E, Toussaint C, *et al.* High-dose ifosfamide: circumvention of resistance to standard-dose ifosfamide in advanced soft tissue sarcomas. *J Clin Oncol* 1995; 13: 1600–8.
44. Patel SR, Vadhan-Raj S, Papadopolous N, Plager C, Burgess MA, Hays C, *et al.* High-dose ifosfamide in bone and soft tissue sarcomas: results of phase II and pilot studies – dose-response and schedule dependence. *J Clin Oncol* 1997; 15: 2378–84.
45. Buesa JM, Lopez-Pousa A, Martin J, Anton A, del Muro JG, Bellmunt J, *et al.* Phase II trial of first-line high-dose ifosfamide in advanced soft tissue sarcomas of the adult: a study of the Spanish Group for Research on Sarcomas (GEIS). *Ann Oncol* 1998; 9: 871–6.
46. Nielsen OS, Judson I, van Hoesel Q, le Cesne A, Keizer HJ, Blay JY, *et al.* Effect of high dose ifosfamide in advanced soft tissue sarcomas. A multicentre phase II study of the EORTC Soft Tissue and Bone Sarcoma Group. *Eur J Cancer* 2000; 36: 61–7.
47. Jaffar Z, Blum RH, Cacavio A, Rosen G. Ifosfamide 14–24 gms/m$^s$ an outpatient study in sarcomas. *Proc Am Soc Clin Oncol* 1998; 17: 511.
48. Tichler T, Ghodsizade E, Brenner H. Failure of continuous infusion high dose ifosfamide as second line treatment for sarcomas: poor response rate and unacceptable renal and C.N.S. toxicity. *Proc Am Soc Clin Oncol* 1999; 18: 544.
49. van Oosterom AT, Mouridsen HT, Nielsen OS, Dombernowsky P, Judson I, Krzemienlecki K, Svancarova L, *et al.* Results of randomized phase II studies of the EORTC SoftTissue and Bone Sarcoma Group (STBSG) with two different ifosfamide regimens in first- and second-line chemotherapy in advanced soft tissue sarcoma patients. *Eur J Cancer* 2002; 18: 2397–406.
50. Lorigan PC, Verweij J, Papai Z, Rodenhuis S, le Cesne A, Leahy M, *et al.* Randomised phase III trial of two investigational schedules of ifosfamide versus standard dose doxorubicin in patients with advanced or metastatic soft tissue sarcoma (ASTS). *Proc Am Soc Clin Oncol* 2002; 21: 405a.
51. Frustaci S, Comandone A, Bearz A, Boglione A, Buonadonna A, Stefanovski P, *et al.* Efficacy and tolerability of an ifosfamide continuous infusion (IFO-C.I.) soft tissue sarcoma (STS) patients (pts). *Proc Am Soc Clin Oncol* 1998; 17: 518.
52. Frustaci S, Buonadonna A, Galligioni E, Favaro D, De Paoli A, Lo Re G, *et al.* Increasing 4′-epidoxorubicin and fixed ifosfamide doses plus granulocyte-macrophage colony-stimulating factor in advanced soft tissue sarcomas: A pilot study. *J Clin Oncol* 1997; 15: 1418–26.
53. Leyvraz S, Bacchi M, Cerny T, Lissoni A, Sessa C, Bressoud A, *et al.* Phase I multicenter study of combined high-dose ifosfamide and doxorubicin in the treatment of advanced sarcomas. *Ann Oncol* 1998; 9: 877–84.
54. Frustaci S, Buonadonna A, Romanini A, Comandone A, Dalla Palma M, Gamucci T, *et al.* Increasing doses of continuous infusion ifosfamide and fixed dose of bolus epirubicin in soft tissue sarcomas. A study of the Italian Group on Rare Tumors. *Tumori* 1999; 85: 229–33.
55. De Pas T, Curigliano G, Masci G, Catania C, Comandone A, Boni C, *et al.* Phase I study of twelve-day prolonged infusion of high-dose ifosfamide and doxorubicin in adult patients with advanced soft tissue sarcomas. *Ann Oncol* 2002; 13: 161–6.
56. Hicks LG, Balcerzak SP, Zalupski M. GM-CSF did not allow doxorubicin dose escalation in the MAID regimen: a phase I trial. A Southwest Oncology Group study. *Cancer Invest* 1996; 14: 507–12.
57. Vadhan-Raj S, Patel S, Burgess M, Papadopoulos N, Plager C, Johnston T, *et al.* Phase II trial of adriamycin (A), ifosfamide (I), mesna (M) uroprotection, dacarbazine (D) (MAID) with PIXY321 (GM-CSF/IL-3 fusion protein) or G-CSF in patients (pts) with soft tissue sarcoma (STS). *Proc Am Soc Clin Oncol* 1996; 15: 525.
58. Patel SR, Vadhan-Raj S, Burgess MA, Plager C, Papadopoulos NE, Jenkins J, *et al.* Results of two consecutive trials of dose-intensive chemotherapy with doxorubicin and ifosfamide in patients with sarcoma. *Am J Clin Oncol* 1998; 21: 317–21.
59. Pronzato P, Losardo P, Pensa F. High-dose-intensity combination chemotherapy for advanced sarcomas: a pilot study. *Cancer Chemo Pharmacol* 1998; 41: 513–6.
60. Reichardt P, Tilgner J, Hohenberger P, Dorken B. Dose-intensive chemotherapy with ifosfamide, epirubicin and filgrastim for adult patients with metastatic or locally advanced soft tissue sarcoma: a phase II study. *J Clin Oncol* 1998; 16: 1438–43.
61. De Pas T, De Braud F, Orlando L, Nole F, Munzone E, Zampino MG, *et al.* High-dose ifosfamide plus adriamycin in the treatment of adult advanced soft tissue sarcomas: is it feasible? *Ann Oncol* 1998; 9: 917–9.
62. Palumbo R, Neumaier C, Cosso M, Bertero G, Raffo P, Spadini N, *et al.* Dose-intensive first-line chemotherapy with epirubicin and continuous infusion ifosfamide in adult patients with advanced soft tissue sarcomas: a phase II study. *Eur J Cancer* 1999; 35: 66–72.
63. Lopez-Pousa A, Buesa JM, Montalar J, Martin J, Maurel J, del Muro JG, *et al.* Phase II trial of doxorubicin and high-dose ifosfamide (DXHDI) in advanced previously untreated soft tissue sarcoma (TS) patients. A study of the Spanish Group for Research on Sarcomas (GEIS). *Proc Am Soc Clin Oncol* 1999; 18: 544.
64. Serrone L, Zeuli M, Gamucci T, Nardi M, Cognetti F. A phase II study of dose-intense ifosfamide plus epirubicin with hematopoietic growth factors for the treatment of patients with advanced soft tissue sarcomas; a novel sequential schedule. *Cancer Chemother Pharmacol* 2001; 47: 206–10.
65. Le Cesne A, Judson I, Crowther D, Rodenhuis S, Keizer HJ, Van Hoesel Q, *et al.* Randomized phase III study comparing conventional-dose doxorubicin plus ifosfamide versus high-dose doxorubicin plus ifosfamide plus recombinant human granulocyte-macrophage colony-stimulating factor in advanced soft tissue sarcomas: a trial of the European Organization for Research and Treatment of Cancer/Soft Tissue and Bone Sarcoma Group. *J Clin Oncol* 2000; 18: 2676–84.

66. Bui NB, Demaille MC, Chevreau C, Blay JY, Cupissol D, Rios M, *et al.* qMAID vs MAID + 25% with G-CSF in adults with advanced soft tissue sarcomas (ST). First results of a randomized study of the FNCLCC Sarcoma Group. *Proc Am Soc Clin Oncol* 1998; 17: 517.
67. Reichardt P, Verweij J, Crowther D. Current controversies in cancer: should high-dose chemotherapy by used in the treatment of soft tissue sarcoma? *Eur J Cancer* 1997; 33: 1351–60.
68. Pinkerton CR. Megatherapy for soft tissue sarcomas. EBMT experience. *Bone Marrow Transplant* 1991; 7: 120–2.
69. Dumontet C, Biron P, Bouffet E, Blay J-Y, Meckenstock R, Chauvin F, *et al.* High dose chemotherapy with ABMT in soft tissue sarcomas: a report of 22 cases. *Bone Marrow Transplant* 1992; 10: 405–8.
70. Kessinger A, Petersen K, Bishop M, Schmit-Pokorny K. High dose therapy (HDT) with autologous hematopoietic stem cell rescue (HSCR) for patients with metastatic soft tissue sarcoma. *Proc Am Soc Clin Oncol* 1994; 13: 480.
71. Blay J-Y, Bouhour D, Ray-Coquard I, Dumontet C, Philip T, Biron P. High-dose chemotherapy with autologous hematopoietic stem-cell transplantation for advanced soft tissue sarcoma in adults. *J Clin Oncol* 2000; 18: 3643–50.
72. Bokemeyer C, Franzke A, Hartmann JT, Schober C, Arseniev L, Metzner B, *et al.* A phase I/II study of sequential, dose-escalated, high dose ifosfamide plus doxorubicin with peripheral blood stem cell support for the treatment of patients with advanced soft tissue sarcomas. *Cancer* 1997; 80: 1221–7.
73. Reichardt P, Tilgner J, Mapara MY, Hohenberger P, Dorken B. Dose-intensive chemotherapy with stem cell support for adult patients with advanced soft tissue sarcoma: a phase I study. *Sarcoma* 1997; 1: 219.
74. Myong H, Rosen G, Forscher C, Hurvitz C, Hamburg S, Qasabian L, *et al.* High dose chemotherapy (CTX) with autologous peripheral stem cell transplantation (PSCT) for advanced sarcoma. *Proc Am Soc Clin Oncol* 1998; 17: 511.
75. Chow W, Somlo G, Doroshow JH, Reardon D, ter Veer A, Leong L, *et al.* Sequential high-dose chemotherapy (HDCT) and peripheral blood progenitor cell rescue (PBPC) in young adults with soft tissue and bone sarcomas (SARC). *Proc Am Soc Clin Oncol* 1998; 17: 512.
76. Falk MH, Salat C, Ochmann O, Knabe H, Mempel W, Sauer HJ, *et al.* High-dose chemotherapy (HDCT) with peripheral blood stem cell rescue (PBSCR) for adults with soft tissue sarcomas (STS). *Proc Am Soc Clin Oncol* 1999; 18: 544.
77. Samuels BL, Gale DM, Michel J, Murray JL, Das Gupta TK, Lad T, *et al.* Dose dense chemotherapy for sarcomas using whole-blood derived peripheral stem cell rescue. *Proc Am Soc Clin Oncol* 1999; 18: 543.
78. Montemurro F, Grosso F, Ferrante P, Rosti G, Aglietta M. High-dose chemotherapy with hematopoietic stem cell transplant (HDCT/HSCT) for soft tissue sarcoma (STS) of the adult: Analysis of the European Group for Blood and Marrow Transplantation (EBMT) Registry. *Proc Am Soc Clin Oncol* 2001; 20: 363a.
79. Falk MH, Hiddemann W, Kolb HJ, Mempel W, Ochmann O, Ostermann H, Salat C, *et al.* High-dose chemotherapy with peripheral blood stem cell rescue as consolidation treatment for adult patients with chemosensitive metastatic soft tissue sarcoma. *Proc Am Soc Clin Oncol* 2001; 20: 361a.
80. Stadtmauer EA, O'Neill A, Goldstein LJ, Crilley PA, Mangan KF, Ingle JN, *et al.* Conventional-dose chemotherapy compared with high-dose chemotherapy plus autologous hematopoietic stem-cell transplantation for metastatic breast cancer. Philadelphia Bone Marrow Transplant Group. *New Engl J Med* 2000; 13: 1069–76.
81. Lotz JP, Cure H, Janvier M, Morvan F, Asselain B, Guillemot M, *et al.* High-dose chemotherapy (HD-CT) with hematopoietic stem cell transplantation (HSCT) for metastatic breast cancer (MBC): results of the French Protocol PEGASE 04. *Proc Am Soc Clin Oncol* 1999; 18: 43.
82. Tuveson DA, Fletcher JA. Signal transduction pathways in sarcoma as targets for therapeutic intervention. *Curr Opin Oncol* 2001; 13: 249–55.
83. Heymach JV. Angiogenesis and antiangiogenic approaches to sarcomas. *Curr Opin Oncol* 2001; 13: 261–9.
84. Maki TG. Soft tissue sarcoma as a model disease to examine cancer immunotherapy. *Curr Opin Oncol* 2001; 13: 270–4.
85. Demetri GD, Fletcher CD, Mueller E, Sarraf P, Naujoks R, Campbell N, *et al.* Induction of solid tumor differentiation by the peroxisome proliferator-activated receptor-gamma ligand troglitazone in patients with liposarcoma. *Proc Natl Acad Sci USA* 1999; 96: 3951–6.
86. Goss G, Demetri G. Medical management of unresectable, recurrent low-grade retroperitoneal liposarcoma: integration of cytotoxic and non-cytotoxic therapies into multimodality care. *Surg Oncol* 2000; 9: 53–9.
87. Demetri GD, Spiegelman B, Fletcher CD, Mueller E, Sarraf P, Naujoks R, *et al.* Differentiation of liposarcomas in patients treated with the PPAR$\gamma$ ligand troglitazone: documentation of biologic activity in myxoid/round cell and pleomorphic subtypes. *Proc Am Soc Clin Oncol* 1999; 18: 535.
88. Presant CA, Bartolucci AA, Lowenbraun S. Effects of amphotericin B on combination chemotherapy of metastatic sarcomas. *Cancer* 1984; 53: 214–8.
89. van der Graaf WTA, Dijk AC, Sleijfer DTh, Hoekstra HJ, de Vries EGE, Willemse PHB, *et al.* Epirubicin, vindesine and ifosfamide (EVI) plus or minus amiodarone in the treatment of soft tissue sarcomas. *Proc Am Soc Clin Oncol* 1996; 15: 525.
90. Bramwell VHC, Morris D, Ernst DS, Hings I, Blackstein M, Venner PM, *et al.* Safety and efficacy of the MDR inhibitor Biricodar (VX-710) with concurrent doxorubicin in patients with anthracycline resistant advanced soft tissue sarcoma. *Clin Cancer Res* 2002; 8: 383–93.
91. Colvin OM. Drug resistance in the treatment of sarcomas. *Semin Oncol* 1997; 24: 580–91.
92. Senderowicz AM, Headlee D, Stinson SF, Lush RM, Kalil N, *et al.* Phase I trial of continuous infusion flavopiridol, a novel cyclin-dependent kinase inhibitor, in patients with refractory neoplasms. *J Clin Oncol* 1998; 16: 2986–99.

93. Demetri GD. ET-743: the US experience in sarcomas of soft tissues. *Anticancer Drugs* 2002; 13 (Suppl 1): S7–9.
94. Le Cesne A, Blay J, Judson I, Van Oosterom A, Verweij J, Radford J, Lorigan P, *et al*. ET-743 is an active drug in adult soft-tissue sarcoma (STS): a STBSG-EORTC phase II trial. *Proc Am Soc Clin Oncol* 2001; 20: 353a.
95. Brain EG. Safety and efficacy of ET-743: the French experience. *Anticancer Drugs* 2002; 13, S22-4.
96. Van Oosterom AT, Judson I, Verweij J, Stroobants S, Di Paola E, Dimitrijevic S, *et al*. Safety and efficacy of imatinib (STI571) in metastatic gastrointestinal stromal tumors (GIST), a phase I study. *Lancet* 2001; 358: 1421–3.
97. Demetri GD, van Mehren M, Blanke CD, Van der Abbeele, Eisenberg B, Roberts PJ, *et al*. Efficacy and safety of imatinib mesylate in advanced gastrointestinal stromal tumors. *New Engl J Med* 2002; 347: 472–80.
98. Benjamin R, Rankin C, Fletcher C, Von Mehren M, Blanke C, Maki R, *et al*. Effectiveness of Imatinib mesylate for metastatic GIST at two dose levels: Phase III Early Results of Sarcoma Intergroup Study S0033. *Proc Am Soc Clin Oncol* 2003; 22: 814.
99. Mitchell G, Thomas JM, Harmer CL. Aggressive fibromatosis: evidence for a stable phase. *Sarcoma* 1998; 2: 149–54.
100. Weiss AJ, Lackman RD. Low-dose chemotherapy of desmoid tumors. *Cancer* 1989; 64: 1192–4.
101. Azzarelli A, Casali P, Fissi S, Gronchi A, Rasponi A, Bertulli R. Effective control of advanced aggressive fibromatosis with chemotherapy. Eight years of experience with methotrexate + vinblastine in 27 patients. *Sarcoma* 1997; 1: 200.
102. Reich S, Overberg-Schmidt US, Buhrer C, Henze G. Low-dose chemotherapy with vinblastine and methotrexate in childhood desmoid tumors. *J Clin Oncol* 1999; 17: 1086–90.
103. Seiter K, Kemeny N. Successful treatment of a desmoid tumor with doxorubicin. *Cancer* 1993; 71: 2242–4.
104. Hamilton L, Blackstein M, Berk T, McLeod RS, Gallinger S, Madlensky L, Cohen Z. Chemotherapy for desmoid tumors in association with familial adenomatous polyposis: a report of three cases. *Can J Surg* 1996; 39: 247–52.
105. Mace J, Biermann JS, Sondak V, McGinn C, Hayes C, Thomas D, Baker L. Response of extraabdominal desmoid tumors to therapy with imatinib mesylate. *Cancer* 2002; 95: 2373–9.
106. Rubin BP, Schuetze SM, Eary JF, Norwood TH, Mirza S, Conrad EU, Bruckner JD. Molecular targeting of platelet-derived growth factor b by imatinib mesylate in a patient with metastatic dermatofibrosarcoma protuberans. *J Clin Oncol* 2002; 20: 3586–91.
107. Casali P, Pastorino U, Bertulli R, Azzarelli A, Quagliuolo V, Fissi S, *et al*. Complete surgery of lung metastases combined with chemotherapy in 31 adult soft tissue sarcomas: long-term results. *Sarcoma* 1997; 1: 196.
108. Hohenberger P, Reichardt P, Tilgner J, Kettelhack C, Schlag PM. Results of combined surgery and high-dose chemotherapy + G-CSF for metastatic or locally advanced soft tissue sarcoma. *Proc Am Soc Clin Oncol* 1999; 18: 542.
109. Wiklund T, Saeter G, Strander H, Alvegard T, Blomqvist C. The outcome of advanced soft tissue sarcoma patients with complete tumor regression after either chemotherapy alone or chemotherapy plus surgery. The Scandinavian Sarcoma Group experience. *Eur J Cancer* 1997; 33: 357–61.
110. Putnam JB, Madden T, Tran HT, Benjamin RS. Isolated single lung perfusion (ISLP) with adriamycin for unresectable sarcomatous metastases. *Proc Am Soc Clin Oncol* 1997; 16: 500.
111. Mavligit GM, Zukwiski AA, Ellis LM, Chuang VP, Wallace S. Gastrointestinal leiomyosarcoma metastatic to the liver. *Cancer* 1995; 75: 2083–8.
112. Bonvalot S, Lecesne A, Terrier P, Le Pechoux C, Elias D, Lasser PH. Preliminary evaluation of complete resection of major sarcomatosis followed by randomization of early post-operative intraperitoneal chemotherapy. *Sarcoma* 1997; 1: 202.
113. Eilber FC, Rosen G, Forscher C, Nelson SD, Dorey FJ, Eilber FR. Surgical resection and intraperitoneal chemotherapy for recurrent abdominal sarcomas. *Ann Surg Oncol* 1999; 6: 645–50.
114. Van Glabbeke M, van Oosterom AT, Oosterhuis JW, Mouridsen H, Crowther D, Somers R. Prognostic factors for the outcome of chemotherapy in advanced soft tissue sarcoma: an analysis of 2,185 patients treated with anthracycline-containing first-line regimens — A European Organization for Research and Treatment of Cancer SoftTissue and Bone Sarcoma Group study. *J Clin Oncol* 1999; 17: 150–7.
115. Rosen G, Forscher C, Lowenbraun S, Eilber F, Eckardt J, Holmes C, Fu YS. Synovial sarcoma: uniform response of metastases to high dose ifosfamide. *Cancer* 1994; 73: 2506–11.
116. Blay J-Y, van Glabbeke M, Verweij J, van Oosterom AT, Le Cesne A, Oosterhuis JW, *et al*. Advanced soft tissue sarcoma: a disease that is potentially curable for a subset of patients treated with chemotherapy. *Eur J Cancer* 2003; 39: 64–9.
117. Reichardt P, Van Glabbeke M, Mouridsen HT, Verweij J, Radford JA, Rodenhuis S, Azzarelli A, *et al*. Recurrent disease is an independent factor associated with inferior survival in patients with advanced soft tissue sarcomas. A retrospective analysis of the EORTC Soft Tissue and Bone Sarcoma Group (STBSG). *Proc Am Soc Clin Oncol* 2001; 20: 354a.
118. Esnaola NF, Rubin B, Baldini E, Vasudevan N, Demetri G.D, Fletcher CD, *et al*. Response to chemotherapy and predictors of survival in adult rhabdomyosarcoma. *Ann Surg* 2001; 234: 215–23.
119. Jameson M, Harvey V, Thompson P, Evans B, Childs J. Juvenile-type tumors in adults carry a grim prognosis. *Proc Am Soc Clin Oncol* 1998; 17: 521.
120. Benjamin RS. Grade 3 nausea, vomiting and myelosuppression or progressive, metastatic sarcoma? *J Clin Oncol* 1987; 5: 833–5.
121. Canellos GP. Myelosuppression and 'conventional' chemotherapy: what price, what benefit? *J Clin Oncol* 1993; 11: 1–2.
122. Edmonson JH. Needed: qualitative improvement in antisarcoma therapy. *J Clin Oncol* 1995; 13: 1531–3.
123. Benjamin RS, Rouesse J, Bourgeois H, van Hoesel QGCM. Current controversies in cancer: Should patients with advanced sarcomas be treated with chemotherapy? *Eur J Cancer* 1998; 34: 958–65.
